# Cupulolithiasis: A Critical Reappraisal

**DOI:** 10.1002/oto2.38

**Published:** 2023-03-01

**Authors:** Olivia Kalmanson, Carol A. Foster

**Affiliations:** ^1^ Department of Otolaryngology University of Colorado Anschutz SOM Boulder Colorado USA

**Keywords:** apogeotropic nystagmus, benign paroxysmal positional vertigo, canalith jam, cupula, cupulolithiasis, otoconia, utricle

## Abstract

**Objective:**

To review the history and pathophysiologic theories for cupulolithiasis and canalith jam in benign paroxysmal positional vertigo.

**Data Sources:**

PubMed, Google Scholar.

**Review Methods:**

Three PubMed and Google Scholar searches were performed, keywords: “cupulolithiasis,” “apogeotropic [and] benign,” and “canalith jam,” resulting in 187 unique full‐text articles in English or with English translation. Figures—Labyrinthine photographs were obtained of fresh utricles, ampullae, and cupulae of a 37‐day‐old mouse.

**Conclusions:**

Freely moving otoconial masses explain most cases (>98%) of benign paroxysmal positional vertigo. Evidence that otoconia adhere strongly or persistently to the cupula is lacking. Apogeotropic nystagmus in the horizontal canal form is often attributed to cupulolithiasis; however, periampullary canalithiasis explains self‐limited nystagmus, and reversible canalith jam explains prolonged apogeotropic nystagmus. Treatment‐resistant cases can be explained by entrapment of particles in the canals or ampullae, but persistent adherence to the cupula remains theoretical.

**Implications for Practice:**

Apogeotropic nystagmus is usually due to freely moving particles and should not be used in studies of horizontal canal benign paroxysmal positional vertigo as the sole method to define entrapment or cupulolithiasis. Caloric testing and imaging may help differentiate jam from cupulolithiasis. Treatment for apogeotropic benign paroxysmal positional vertigo should include maneuvers that rotate the head through 270° to fully clear the canal of mobile particles, using mastoid vibration or head shaking if entrapment is suspected. Canal plugging can be used for treatment failures.

Cupulolithiasis is defined by otoconia that adhere persistently to the cupula, rendering it gravity sensitive. In 1969, Schuknecht first postulated cupulolithiasis as the cause of classic posterior canal benign paroxysmal positional vertigo (P‐BPPV). The paper showed basophilic deposits on postmortem fixed sections of the cupulae in 2 BPPV patients.^1^ In 1962 and 1974 figures, he showed otoconia attached to the utricular side of the cupula, causing a utriculopetal displacement of the cupula on Dix Hallpike,^2^ although by his 1969 text he understood that the characteristic nystagmus of P‐BPPV must stem from utriculofugal displacement. The well‐known fatiguability and short duration of P‐BPPV nystagmus contradict persistent attachment to the cupula, so he proposed that nystagmus resolved when particles detached and recurred as they reattached.^2^ In 1979, Hall et al theorized an alternative explanation, that classic P‐BPPV was caused by particles freely moving in the posterior semicircular canal (SCC), correctly explaining the direction and time course of the accompanying nystagmus.^3^ However, they continued to offer cupulolithiasis as a mechanism for cases with prolonged rather than paroxysmal nystagmus. Epley also adopted canalithiasis as causative in 1980^4^ and then developed the Epley maneuver, the success of which confirmed the free particle theory.^5^ The presence of cupular deposits in postmortem preserved temporal bones was shown to lack correlation with clinical symptoms,^6^, ^7^ and mobile particles were observed and photographed during surgery.^8^, ^9^ Together these findings further substantiated that particles were freely moving and that the cupulolithiasis mechanism for P‐BPPV was incorrect.^3^, ^5^ In 1985, McClure expanded canalithiasis to include the horizontal canal (H‐BPPV).^10^ Canalithiasis was thus gradually accepted as the cause of most cases of BPPV. However, cupulolithiasis continued to be cited but the pathophysiology was altered to persistent rather than paroxysmal positional vertigo.^11^

In 1995, Baloh et al published the first English case report of non‐fatiguing apogeotropic H‐BPPV, reintroducing cupulolithiasis as a mechanism for this form of direction‐changing positional nystagmus (DCPN).^12^ Particles were diagrammed adherent to the utricular side of the cupula. A year later, Nuti et al showed that apogeotropic H‐BPPV could occur if freely moving particles were located in the periampullary canal.^13^, ^14^ This work had been published in the Italian literature prior to Baloh's paper but was overlooked due to the language barrier. Through the work of Gufoni, Asprella‐Libonati, and Vannuchi, who developed new repositioning maneuvers based on periampullary canalithiasis, the concept began to spread by the early 2000s and is now widely cited.^15^, ^16^, ^17^, ^18^, ^19^, ^20^ Since 1996 there are thus two competing pathophysiologies for apogeotropic nystagmus: cupulolithiasis and periampullary canalithiasis. The latter was slower to be adopted than cupulolithiasis, and even today, many researchers treat apogeotropic nystagmus as equivalent to cupulolithiasis,^21^ although the Barany society definitions include both.^22^

Clinical signs of cupulolithiasis are not fixed but have been redefined three times, first from classic BPPV with paroxysmal nystagmus,^1^ to nonfatiguing positional nystagmus,^3^ and then to apogeotropic nystagmus.^12^ Treatment failures are more common in apogeotropic cases of H‐BPPV than in canalithiasis‐caused geotropic forms. Some propose that treatment resistance suggests cupulolithiasis, presumably because particles firmly attach to the cupula and cannot be dislodged easily.^16^, ^23^, ^24^, ^25^ Moreover, since both sides of the cupula should be equally adherent to particles, the concepts of utricular‐side and canal‐side cupulolithiasis have proliferated,^23^, ^26^ and new maneuvers have been developed in response.^27^, ^28^

A third mechanism of BPPV, canalith jam, is a complication of canalithiasis first described by Epley.^29^ The concept was not reported by others until after his 2001 paper^30^ and was only occasionally mentioned for several years. He postulated previously freely moving particles became entrapped at natural narrowings in the canal, partially or completely occluding it. The clinical signs of jam he outlined were prolonged direction‐fixed nystagmus, canal paresis, and impaired vestibular head impulse test (VHIT) responses. Von Brevern further clarified the mechanism in 2001, when he reported a case of jam and attributed the prolonged spontaneous nystagmus to tonic deviation of the cupula caused by the jam‐induced canal plugging.^31^ While the original signs of jam seemed to differentiate it from cupulolithiasis, the lines have blurred over time. Like cupulolithiasis, jams are often resistant to maneuvers, and several cases of apogeotropic, treatment‐resistant H‐BPPV secondary to mobile jams have been reported in the last few years.^17^, ^32^, ^33^, ^34^ The overlap between periampullary canalithiasis, canalith jam, and cupulolithiasis raises uncertainty about how to meaningfully define these entities.

The objective of this review is to explore the history, pathophysiologic theories, and physical evidence underlying cupulolithiasis and canalith jam. We concentrate on the HSSC, which is the canal involved in most articles on these subjects, but all three canals should follow similar mechanisms.

## Methods

**Figure 1:**
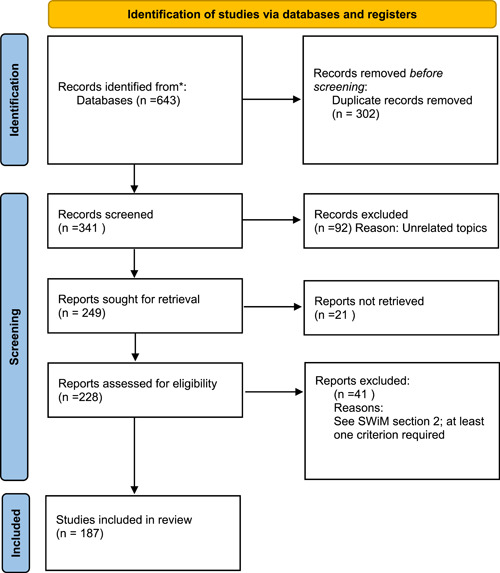
PRISMA flow chart.

Syntheses without metanalysis (SWiM) guidelines^35^ recommended by PRISMA were utilized:

(1) Grouping studies for synthesis:

Three Pubmed and Google Scholar searches were performed: “cupulolithiasis,” “apogeotropic [and] benign,” and “canalith jam”. See Figure 1 for a PRISMA flow chart. One hundred and eighty‐seven unique full‐text articles were identified in English or with English translation from 1969 to the present.

(2) Describe the standardized metric and transformation methods used:

Metanalysis could not be performed because of the heterogeneity of the articles sampled, and no standardized metric was required. Data collected for all studies:

Canal affected;

Nystagmus type;

BPPV mechanisms and particle locations;

Canal paresis;

Nystagmus velocity profiles

(3) Criteria used to prioritize results for summary and synthesis.

The study sought all articles that met criteria in the first section and that provided relevant data from the second section.

(4–6) Investigation of heterogeneity in reported effects, certainty of evidence, and data presentation methods: These are quantitative measures that do not apply to descriptive reviews.

For our figures, murine cupula and otoconia were obtained as follows: Pairs of fresh utricles, saccules, and semicircular canal ampullae were harvested in a pool of phosphate‐buffered saline from a 37‐day‐old C57BL/6J mouse after being deeply anesthetized and decapitated. Photographs were taken on a Leica M165FC stereoscope. Animal work was approved by the Institutional Animal Care and Use Committee of the University of Colorado campus (Protocol #96).

## Discussion

### Literature Review

We reviewed 187 papers published from 1969 to April 2022, 114 (61%) of which were published in the last decade. The majority of these discuss H‐BPPV (148/187 [79%]).

Most reviewed papers published in the last decade (64/114 [56%]) report cupulolithiasis and canalithiasis as the causes of BPPV without mentioning that canalithiasis predominates or that canalith jam is an alternate mechanism. The remaining 50/114 (44%) discuss canalithiasis as the primary mechanism, and only 12/114 (10.5%) mention canalith jam.

The nomenclature of the SSCs and BPPV is not standardized and is confusing. For example, *short arm* sometimes refers to the ampullary‐utricular opening, and at other times to the ampullary neck leading into the canal. We use *periampullary canal* to describe the portion of the semicircular canal opening into the ampulla, and *canal opening* for the portion opening to the utricle. The ampulla and cupula have a *utricular side* that opens into the utricle, and a *canal side* that opens into the semicircular canal. Table 1 contains a listing of various definitions used in this article for clarity.

**Table 1:** Nomenclature Variations

Canal anatomy	Alternate names	Definition
Horizontal (Comparative anatomy term)	Lateral (human surgical anatomy term)	Horizontally‐oriented canal placed laterally in the skull
Posterior	Inferior	Vertically oriented canal, posteroinferior in skull
Anterior	Superior	Vertically oriented canal, anterosuperior in skull
Canal opening	Nonampullary arm or tract; utricular arm; posterior arm	Opening of common crus or horizontal SSC into the utricular sac
Periampullary canal	Anterior arm, short arm, ampullary arm	Section of SSC opening into ampulla
Ampulla anatomy		
Canal side	Ampullary segment or neck; Cup‐C	Side of cupula and ampulla tapering into canal
Utricular side	Short arm; utricular arm; Cup‐U; forearm	Side of cupula and ampulla opening widely into utricle
By nystagmus		
Geotropic	Light cupula	Fast phase beating toward the ground in DCPN
Apogeotropic	Ageotropic; heavy cupula	Fast phase beating away from the ground in DCPN
Null point	Zero plane	During head rotation, horizontal point, or zone in degrees from midline at which DCPN falls to zero and/or reverses direction
Fluid/particle motion	Cupular motion	
Ampullopetal	Utriculopetal	Motion of fluid or particles toward the cupula/ampulla: cupula bulging toward utricular side of ampulla
Ampullofugal	Utriculofugal	Motion toward the canal away from the cupula/ampulla; cupula bulging toward canal side of ampulla

### Anatomy and Physiology of the Cupula, Ampulla, and Utricle

**Figure 2:**
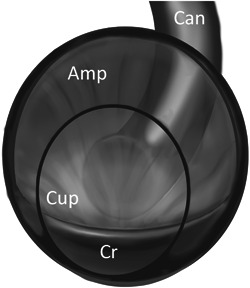
Diagram of human SSC ampulla. The ampulla (Amp) is viewed from the utricular opening nearly perpendicular to the cupula (Cup) and crista (Cr). The canal (Can) arcs away behind the ampulla.

Each SCC is a fluid‐filled truncated torus with a crista/cupular complex dividing the ampulla (Figure 2). It is designed to detect head rotation in which fluid and particles are constrained to move in exactly 2 directions and give rise to 2 different opposing eye movements, resulting in DCPN.

**Figure 3:**
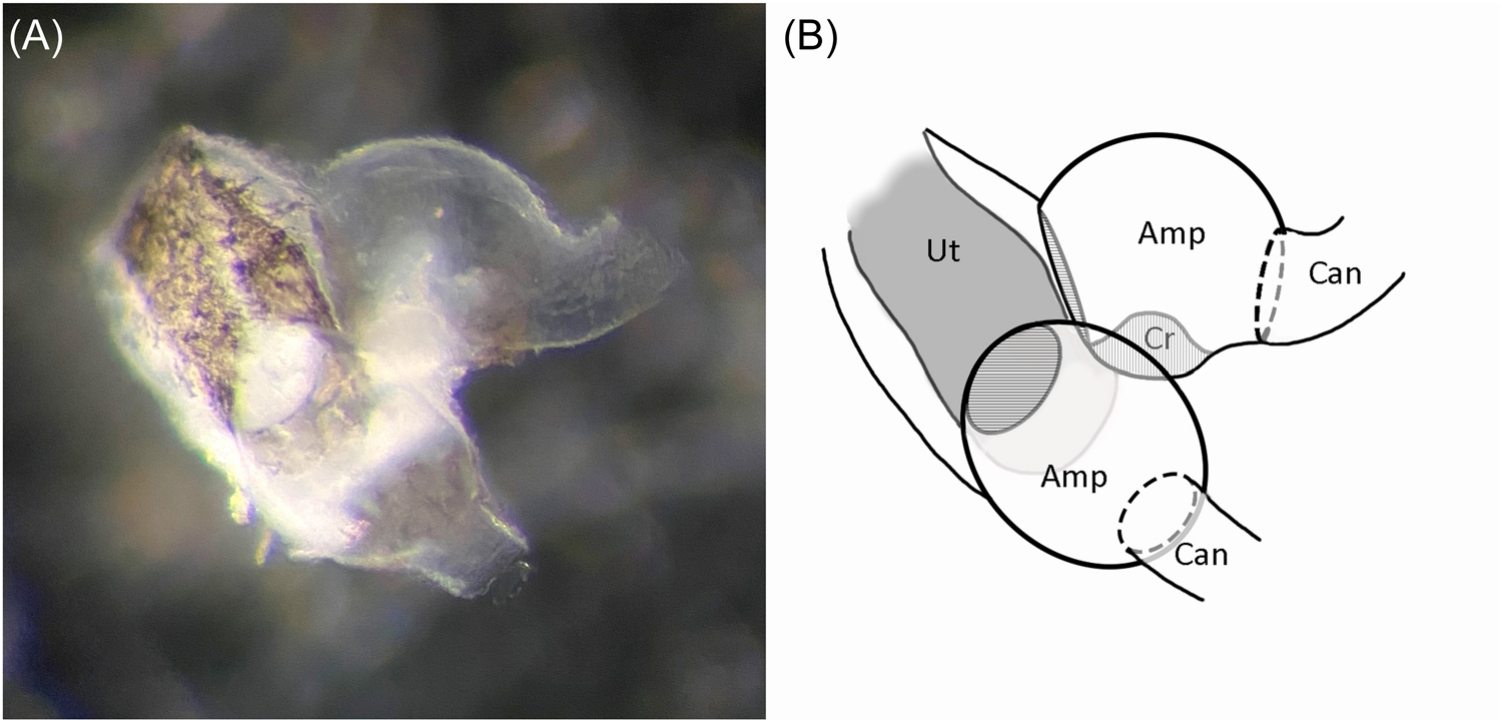
Relationship of ampullary‐utricular openings to utricle. (A) Photograph: 2 murine SSC ampullae and utricle. (B) Diagram: Amp, ampullae; Can, SSCs; CR, crista of the upper ampulla is seen in cross section; Ut, utricle.

The cupula is a flexible, transparent fibrillar membrane sealed to the ampullary wall that is easily distended by fluid motion.^36^ Its major component is an elastic glycoprotein, cupulin.^37^ The narrow diameter of the canal with respect to the larger ampulla increases the syringe‐like effect of fluid motion in the canal directed against or away from the center of the cupula.^36^ The utricular side of all three ampullae open widely into the utricular sac (Figure 3). As a result, there is no plunger effect on the utricular side and, importantly, the cupula on that side is constantly exposed to any detached otoconia in the sac.

The utricle is a gravity‐sensing structure lying against the bony wall of the labyrinth, capped by a dense meshwork membrane that embeds the otoconia.^38^ The major glycoprotein component, otolin, is intensely adherent, and covers the otoconia themselves with interconnecting fibrils.^38^, ^39^ Elasticity is not a requirement of the membrane and attached otoconia because they shift as a plate rather than stretch. In spite of their strong adherence to the utricle, the otoconia and fragments of the associated membrane frequently become detached, leading to BPPV. Pieces of detached membrane and otoconia clump with other fragments to form masses large enough to span the canal.^9^, ^17^

### Direct Evidence Supporting Cupulolithiasis

Pathologic evidence for cupulolithiasis was provided in Schuknecht's paper.^1^ Two slides showed basophilic material attached to the apical surface of the posterior canal cupula in 2 patients with classic P‐BPPV. The cupulae were detached from the ampullary walls and retracted onto the crista. These slides are often listed as proof of cupulolithiasis. However, there is strong evidence against this. First, the 2 patients had classic, canalithiasis‐type BPPV, so the presence of material on the cupula is unexplained. Second, the presence of recurrent BPPV prior to death would require that both patients had intact cupulae attached to the ampullary wall. Cupulae that are detached from the ampulla lose the ability to signal movement,^40^ so a continued history of BPPV goes against any premortem detachment. Basophilic material on cupulae is frequent in patients without a history of BPPV,^6^ also suggesting that this is a postmortem artifact. Therefore, the specimens provided by Schuknecht do not prove that cupulolithiasis occurs.

Animal models of cupulolithiasis provide some pathologic evidence favoring the existence of cupulolithiasis. When a mass of otoconia is dropped onto a horizontal cupula, it causes a paroxysmal burst of output at impact, followed by a stabilization at a much lower and persistent level.^41^ This is similar to a person jumping onto a trampoline, indenting it, then standing without moving so the trampoline returns to a less‐indented steady state. If particles adhere to the cupula, then after initial placement only the steady state form would be detectable.

Placing otoconia on a horizontally oriented cupula has been shown to result in persistent low‐grade action potentials. The force has been calculated as 1/15th of the force exerted by canalithiasis.^42^ Cupular action potentials in response to rotation are suppressed in a cupulolithiasis model.^43^ Mathematical models of cupulolithiasis also support low‐grade nystagmus.^44^ Overall, these models suggest that cupulolithiasis should manifest as a short latency, low‐grade, persistent/nonparoxysmal DCPN.

### Are the Otoconia Uniquely Adherent to the Cupula?

The otoconia are covered with an adhesive substance^45^ and tend to adhere to the otolith organs, instruments, and epithelial surfaces. Cupulolithiasis theory demands that the otoconia adhere strongly and persistently (for days or weeks) to the cupula, explaining the frequently noted resistance to treatment with maneuvers in this group.^25^, ^46^ Because the H‐SCC is the most frequently affected, if cupulolithiasis exists the adherence must be strong enough to resist gravity, because the cupula in this canal is near vertically oriented in most upright head positions,^47^ continuously exposing any particles on the cupula to gravitational dislodgement. There is no obvious evolutionary advantage for the cupula to be sticky or uniquely adherent to otoconia, since it is a fluid motion sensor that is impaired if it develops a gravity‐sensing function.

Otoconia often adhere briefly to the canal walls.^30^ Patients with BPPV can lack nystagmus on the first Dix Hallpike, with a strong response on the second, suggesting that particles were temporarily lodged in the canal.^48^ If a first Dix Hallpike is negative, tapping or applying vibration to the mastoid can cause a burst of nystagmus indicating that particles are mobilizing after temporary adherence to the canal wall.^49^ No studies have compared the relative adhesiveness of various surfaces in the membranous labyrinth other than the otolith organs. The surface area of the cupula is much less than that of the canal, so if all surfaces are assumed to be equally adherent, it is less likely to be the site of adherence.

Although both the cupula and the otoconial membrane have a gelatinous protein structure, the otoconial membrane has much greater adhesiveness, requiring 15 minutes of drilling to remove particles from half of the specimens.^50^ A few authors have created bullfrog models of cupulolithiasis by applying otoconia directly to the cupula.^43^, ^51^, ^52^ The duration of recording in these studies is seconds to minutes only, and attachments for >30 minutes have not been documented experimentally. Otoconia applied to the cupula were released by vibration in 2 minutes on average in 10/10 trials and gravity alone detached them in 2/10 trials.^50^ Canalith jams were also noted indicating otoconia adhere to canal walls.^43^ Comparisons of the duration or tenacity of adherence to the cupula versus canal wall, particularly during high acceleration head movements typical of normal motion, have not been explored.

Elastic membranes similar to the cupula may have self‐cleansing properties. This can be seen with a trampoline in winter, which can accumulate a thick, embedded ice layer. A single step deforms it enough that the ice can be fractured and detached over the entire mat. The same elastic recoil in the springs and mat is responsible for launching the trampolinist into the air. Like a trampoline, the cupula flexes constantly.^36^, ^53^ Rigid substances (like otoconia) may adhere to it, but as the cupula recoils elastically, inflexible masses should fracture off. Another factor in self‐cleansing is the intermittent jet of endolymph from the canal striking the middle of the cupula,^36^, ^53^, ^54^ which would tend to drive particles on the cupula toward the periphery.

In summary, while otoconia can attach to the cupula at least transiently, no research has proven that the cupula is more attractive to otoconia than other labyrinthine surfaces, or that they can remain attached for more than 30 minutes. Additional research comparing the adherence and elastic properties of the cupula, otoconia, and otolithic membrane is needed.

### Is Apogeotropic Nystagmus Equivalent to Cupulolithiasis?

For H‐BPPV, geotropic nystagmus is widely ascribed to canalithiasis, with particles located in the half of the canal nearest the canal opening.^10^, ^13^, ^22^, ^55^ In contrast, the majority of papers on apogeotropic H‐BPPV (89/146 [61%]) equate all apogeotropic nystagmus with cupulolithiasis.^56^ However, particles moving freely in the periampullary canal have also been shown to cause apogeotropic nystagmus^13^, ^14^, ^16^, ^22^, ^57^, ^58^, ^59^, ^60^, ^61^ and it is included in the Barany Society H‐BPPV definition.^22^ As recently as 2021, 15 articles discussed apogeotropic nystagmus, but 1/3 failed to discuss periampullary otoconia as a possible cause. This raises the possibility that alternative diagnoses to cupulolithiasis may not have been appropriately considered in these studies.

A review of all H‐BPPV papers with data on apogeotropic versus geotropic cases to the present with ≥10 patients (20 papers, 2856 patients) resulted in a proportion of 41% apogeotropic to 59% geotropic cases. Since H‐BPPV is about 10% of all BPPV, the apogeotropic form accounts for about 4% of all BPPV. Three papers provide indirect data on the proportion of the apogeotropic group attributable to periampullary canalithiasis, since this type is generally not treatment resistant if maneuvers that clear the entire H‐SSC are used. Martellucci et al successfully resolved 119/134 (88.8%) H‐BPPV cases with maneuvers designed to treat periampullary canalithiasis. Of the apogeotropic cases, 42/54 (78%) resolved, leaving 12/54 (22%) as possible cupulolithiasis or jam.^46^ Asprella Libonati reported 47/73 (65%) apogeotropic H‐BPPV resolved and 26/73 (35%) required multiple maneuvers with 1/73 failures (2%).^14^ Maas et al reported resolution in 25/40 (63%) apogeotropic H‐BPPV cases and required additional treatments in 15/40 (37%).^16^

Periampullary canalithiasis thus likely caused 114/167 cases (mean 68%, range 63%‐78%) of apogeotropic H‐BPPV in this series, leaving cupulolithiasis and/or canalith jam to account for 53/167 (mean 31%, range 22%‐37%) of apogeotropic and treatment‐resistant BPPV. Since apogeotropic cases represent about 4% of all BPPV,^46^ cupulolithiasis and/or jam are rare and account for only about 1% of all BPPV. Further studies are needed to narrow these estimates, and all studies of apogeotropic BPPV need to attempt differentiating periampullary canalithiasis from reversible jam or cupulolithiasis.

### Does Treatment Resistance Differentiate Cupulolithiasis From Jam?

Many use cupulolithiasis to explain cases whose symptoms cannot be resolved with maneuvers,^47^ but this is equally true for canalith jam.^33^ Cupulolithiasis theory suggests that otoconia adhere so strongly to the cupula that they cannot be detached, while in jam a plug is blocked from exiting the canal due to its size and shape.^45^, ^62^

Because apogeotropic H‐BPPV is often incorrectly attributed to cupulolithiasis, some of the treatment resistance is due to failure to fully treat periampullary canalithiasis. Like apogeotropic H‐BPPV, P‐BPPV is an example of periampullary BPPV; particles are located just outside the ampulla, but Epley's maneuver is able to remove them because it uses gravity and rotation in the plane of the canal through 270°, the full length of the canal.^30^

Maneuvers for apogeotropic H‐BPPV, in contrast, are not as effective as maneuvers for P‐BPPV.^46^, ^63^ First, the head is not always rotated in the plane of the canal (eg, barbecue rotations). It is technically difficult to rotate in the plane of the canal, which requires supine positioning with the head elevated 30°, then prone with the head tilted downward 30°. Second, many maneuvers include rotations that are less than 270°.^14^, ^64^ These are ideal for geotropic nystagmus, when particles are near the canal opening, but cannot move particles easily from the periampullary canal through the entire HSSC to the opening.^65^ In these cases, an additional maneuver, such as the inverted Gufoni maneuver, is added to rotate particles from deep in the canal to nearer the opening first (termed geotropic conversion).^64^, ^66^, ^67^ Maneuvers do not actually convert the disorder to a new form, but merely move particles from the section proximal to the ampulla to the section distal to it in the same canal. Because periampullary canalithiasis requires additional maneuvers to treat, failure to use these maneuvers may result in over‐estimation of the frequency of treatment‐resistant H‐BPPV.

All studies of apogeotropic H‐BPPV need to include maneuver sequences that fully clear the periampullary canal. Treatment resistance, however, cannot differentiate jam from cupulolithiasis since in both cases, particles are entrapped in the canal or ampulla.

### Reversible Canalith Jam Causes an Identical Apogeotropic Nystagmus

**Figure 4:**
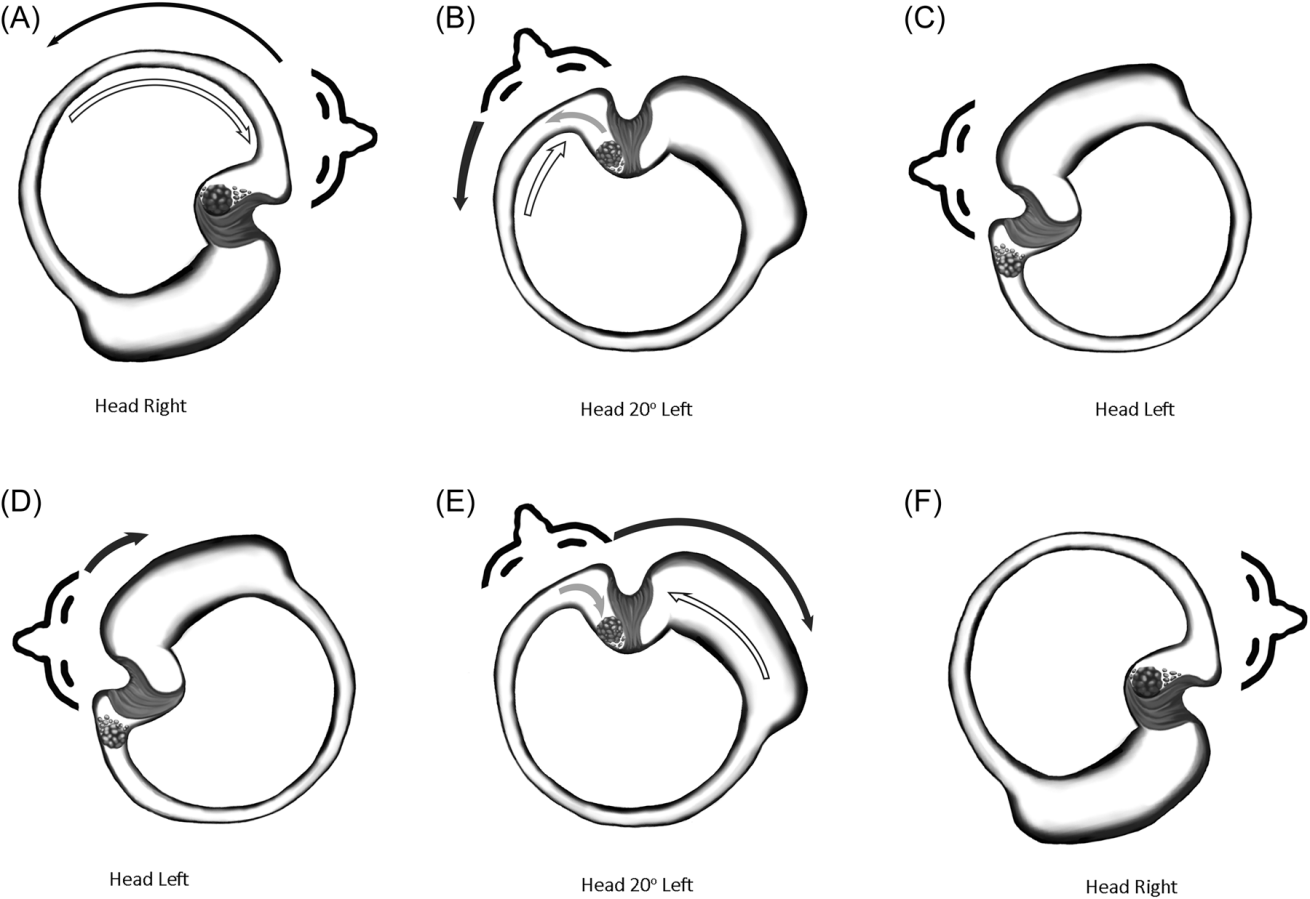
Mechanism of reversible canalith jam.^25^ View: Axial from vertex, perpendicular to left H‐SCC. Arrows indicate upcoming motion. (A–C) Leftward head turn. (D–F) Rightward head turn. Black, head; C, cupula; gray, otoconia; H‐SCC, horizontal semicircular canal; Ut, utricle; white, endolymph.

Canalith jam occurs when particles become entrapped at narrowings or bends in the canal or at the ampullary neck^29^, ^30^, ^31^, ^33^ (Figure 4). Because otoconia clump together, they can pass individually through the canal toward the cupula but can then form an irregular clump in the larger ampulla that exceeds the canal diameter at one of these points, preventing their exit.^17^, ^68^ When the jam is lodged in place, fixed cupular deflection occurs from the negative pressure between the jam and the cupula, resulting in severe vertigo and prolonged spontaneous, direction‐fixed nystagmus.^30^

However, the otoconial masses in jam are not invariably fixed. Epley noted that a jam could be dislodged by rotating the head opposite to the direction causing the jam.^30^ A number of authors have noted that the jam can move back and forth between the cupula and a narrowing in the canal or at the ampullary neck.^15^, ^17^, ^22^, ^33^, ^34^, ^69^ These reversible jams result in a different clinical presentation than a fixed jam. The mobile plug can act as a check ball valve in the canal and cause an apogeotropic DCPN and vertigo symptoms identical to that usually ascribed to cupulolithiasis.^33^, ^34^

Null points during head roll testing in apogeotropic DCPN have been used as evidence for cupulolithiasis because they occur when the cupula is earth‐vertical and therefore not affected by gravity.^70^ However, they can also occur in reversible canalith jam (Figure 4). During a supine roll test starting with the affected ear down, nystagmus is present due to the canal jam deflecting the cupula with negative pressure. As the head is rotated away from the affected ear, the clump of particles dislodges and moves into the ampulla, relieving the negative pressure and cupular deflection, which halts the nystagmus. As head‐turning continues the clump moves onto the cupula, deflecting it in the opposite direction, and restarting nystagmus.^33^ This corresponds to the same location as null points in cupulolithiasis, which are ipsilateral and at about 20° from the midline for H‐BPPV.^47^, ^71^, ^72^, ^73^ Thus, both canalith jam and cupulolithiasis cause apogeotropic DCPN, and null points do not differentiate them.

### Do Nystagmus Profiles Differentiate Them?

Some authors suggest looking for a prolonged time constant or persistent DCPN to differentiate cupulolithiasis from canalithiasis.^70^, ^74^ Particles attached to the cupula should cause a continuous DCPN of long duration, since adaptation to a constant nystagmus is slow. Jam, however, also results in a prolonged DCPN.^33^

In animal models of cupulolithiasis, DCPN is continuous and without a crescendo‐decrescendo peaked pattern.^51^, ^52^ The nystagmus pattern in reversible jams shows a peaked pattern because particles freely move in the canal before jamming,^33^ a pattern confirmed in bullfrog models of canalithiasis.^51^, ^52^ A peaked pattern is shown in figures of purported cupulolithiasis in the literature,^75^ including the very first report of H‐SCC cupulolithiasis,^12^ suggesting that these likely represented periampullary canalithiasis or reversible jam and not cupulolithiasis. Both of these human studies defined cupulolithiasis as apogeotropic nystagmus. Baloh, the senior author of the report that first suggested apogeotropic nystagmus was due to cupulolithiasis,^12^ now feels that most if not all apogeotropic nystagmus is due to periampullary canalithiasis and that cupulolithiasis is primarily of historical interest.^76^

Others have noted that the nystagmus of cupulolithiasis should be lower velocity than with freely moving particles, which have a mechanical advantage in moving the cupula due to a plunger‐like effect.^44^, ^51^, ^65^, ^77^ Case studies of jam show peak velocities exceeding 50 d/s, as high as those found with geotropic/canalithiasis H‐BPPV,^33^ and nystagmus velocities as high as 80°/s have been reported,^78^ which is not consistent with experimental models of cupulolithiasis in which peak velocities are 1/3 those of canalithiasis.^44^

### Does Canal Paresis Indicate Jam?

Canal paresis is often used to distinguish jam from cupulolithiasis.^31^, ^79^, ^80^, ^81^, ^82^ This paresis has been noted to range from partial to complete, suggesting that the blockage can be sieve‐like, allowing some caloric response and a gradual decline in nystagmus over minutes.^30^ Canal paresis has been reported in 37% to 50% of cases of apogeotropic H‐BPPV,^81^, ^82^ which exceeds the 33% of apogeotropic H‐BPPV that are treatment‐resistant,^14^, ^16^, ^46^ however, correlations between treatment resistance and canal paresis have not yet been explored.

If canal paresis reliably indicates jam, this means much of what has passed for cupulolithiasis in the literature is actually jam. Some models, however, suggest that a low‐grade canal paresis is theoretically possible in cupulolithiasis. In a bullfrog model, placement of otoconia on the cupula resulted in a decrease in responsiveness to motion regardless of head position due to increased cupular mass, but this also occurred with jamming of particles in the canal.^43^

There may be ways to differentiate whether a canal paresis is due to jam or cupulolithiasis. In reversible jam, the degree of canal paresis should vary with head position, being least at the null point, intermediate when particles lie on the cupula, and greatest when the canal is plugged. If the head is held for a prolonged time in the canal‐plugged position, the canal paresis and nystagmus may decline due to leakage around or displacement of the jam, while these findings may continue indefinitely in cupulolithiasis. It will also be necessary to screen out other causes of canal paresis, such as adaptation to chronic vertigo, prior vestibular injury, or light cupula.^83^ Further research is needed to explore these potential differences.

### Are There Other Ways to Differentiate Jam and Cupulolithiasis?

Micro‐computerized tomography (CT) supports jam theory by demonstrating filling defects—and possibly blockage—within the canal and ampulla of BPPV patients.^84^ This finding was also noted in nondizzy subjects, however.^32^ Repeat imaging in different head positions may allow detection of mobile, reversible jams. This may represent an objectively verifiable way to differentiate jam from cupulolithiasis in some patients. The images cannot yet determine if particles in the ampulla are attached to the cupula.

In summary, evidence that the otoconia adhere preferentially or for prolonged periods to the flexible cupula is lacking in animal models. The majority (>98%) of H‐BPPV is due to canalithiasis, and apogeotropic nystagmus due to periampullary canalithiasis has been incorrectly attributed to cupulolithiasis. Treatment resistance in H‐BPPV may result from failure to clear the periampullary canal of freely moving particles, while other cases may be explained by canalith jam. Reversible jams cause apogeotropic and prolonged nystagmus with null points indistinguishable from those theorized for cupulolithiasis. CT imaging of filling defects in the canal, the presence of variable canal pareses, and crescendo‐decrescendo nystagmus profiles suggest reversible jam rather than cupulolithiasis. We believe the mechanism of BPPV is canalithiasis with occasional jamming of entrapped particles. A second entrapment mechanism, attachment of otoconia to the cupula, may not be necessary to explain treatment resistance or prolonged nystagmus. Animal models suggest that a persistent, low‐grade DCPN should be the clinical manifestation of cupulolithiasis, and examples of this should be sought. Because of this extensive overlap in findings, we suggest using the term *BPPV with entrapment* for treatment‐resistant cases, which does not require specifying whether particles adhere to the cupula or are blocked from exiting the canal.

### Limitations of the Study

Literature searches rely upon the breadth of studies already done to provide insights. Pathologic specimens for these conditions are rare, and animal models are limited in number, so reliance on clinical studies that did not often differentiate these conditions fully may have affected our analysis. Our study uses English language sources, and important works in other languages may have been overlooked.

### Implications for Practice

Apogeotropic nystagmus is not equivalent to cupulolithiasis, and efforts should be made in studies to clearly differentiate freely moving from entrapped particles. All reported cases of entrapment should include caloric testing to attempt to differentiate jam from cupulolithiasis. Micro‐CT may prove to be a useful modality for diagnosis. Treatment for the apogeotropic form of horizontal canal BPPV should include maneuvers that rotate the head through 270° to fully clear the canal of mobile particles.^58^, ^85^ If jam is suspected, mastoid vibration,^66^ vigorous acceleration/deceleration maneuvers such as head shaking ^86^ or forced prolonged positioning^25^ can be used. Surgical canal plugging should be reserved for treatment failures.^33^

## Author Contributions

**Olivia Kalmanson**, dissection, photo preparation, literature review, writing and editing; **Carol A. Foster**, literature review, illustration, writing and editing.

## Disclosures

### Competing interests

None.

### Funding source

None.
